# Update on the pathogenesis and genetics of Paget’s disease of bone

**DOI:** 10.3389/fcell.2022.932065

**Published:** 2022-08-12

**Authors:** Luigi Gennari, Domenico Rendina, Daniela Merlotti, Guido Cavati, Christian Mingiano, Roberta Cosso, Maria Materozzi, Filippo Pirrotta, Veronica Abate, Marco Calabrese, Alberto Falchetti

**Affiliations:** ^1^ Department of Medicine Surgery and Neurosciences, University of Siena Italy, Siena, Italy; ^2^ Department of Clinical Medicine and Surgery, Federico II University, Naples, Italy; ^3^ Department of Medical Sciences, Azienda Ospedaliera Universitaria Senese, Siena, Italy; ^4^ Unit of Rehabilitation Medicine, San Giuseppe Hospital, Istituto Auxologico Italiano, Piancavallo, Italy; ^5^ Age Related Diseases Unit, Division of Genetics and Cell Biology, San Raffaele Scientific Institute, Milano, Italy; ^6^ Experimental Research Laboratory on Bone Metabolism, Istituto di Ricovero e Cura a Carattere Scientifico (IRCCS), Istituto Auxologico Italiano, Milano, Italy

**Keywords:** Paget’s disease of bone, osteoclast (OCs), genetics, viral inclusion, environmental factors

## Abstract

Studies over the past two decades have led to major advances in the pathogenesis of Paget’s disease of bone (PDB) and particularly on the role of genetic factors. Germline mutations of different genes have been identified, as a possible cause of this disorder, and most of the underlying pathways are implicated in the regulation of osteoclast differentiation and function, whereas other are involved in cell autophagy mechanisms. In particular, about 30 different germline mutations of the *Sequestosome 1* gene (*SQSTM1*) have been described in a significant proportion of familial and sporadic PDB cases. The majority of *SQSTM1* mutations affect the ubiquitin-binding domain of the protein and are associated to a more severe clinical expression of the disease. Also, germline mutations in the *ZNF687* and *PFN1* genes have been associated to severe, early onset, polyostotic PDB with increased susceptibly to neoplastic degeneration, particularly giant cell tumor. Mutations in the *VCP* (Valosin Containing Protein) gene cause the autosomal dominant syndrome “Inclusion Body Myopathy, PDB, Fronto-temporal Dementia,” characterized by pagetic manifestations, associated with myopathy, amyotrophic lateral sclerosis and fronto-temporal dementia. Moreover, germline mutations in the *TNFRSF11A* gene, which encodes for RANK, were associated with rare syndromes showing some histopathological, radiological, and clinical overlap with PDB and in two cases of early onset PDB-like disease. Likewise, genome wide association studies performed in unrelated PDB cases identified other potential predisposition genes and/or susceptibility loci. Thus, it is likely that polygenic factors are involved in the PDB pathogenesis in many individuals and that modifying genes may contribute in refining the clinical phenotype. Moreover, the contribution of somatic mutations of *SQSTM1* gene and/or epigenetic mechanisms in the pathogenesis of skeletal pagetic abnormalities and eventually neoplastic degeneration, cannot be excluded. Indeed, clinical and experimental observations indicate that genetic susceptibility might not be a sufficient condition for the clinical development of PDB without the concomitant intervention of viral infection, in primis paramixoviruses, and/or other environmental factors (e.g., pesticides, heavy metals or tobacco exposure), at least in a subset of cases. This review summarizes the most important advances that have been made in the field of cellular and molecular biology PDB over the past decades.

## 1 Introduction

Paget’s disease of bone (PDB) is a chronic focal disorder of bone remodeling, affecting one (monostotic form) or more bones (polyostotic form) which are typically enlarged and deformed. The affected skeletal sites are generally asymmetric and most frequently include the pelvis, spine, skull, femur and tibia (one or more of these skeletal sites are affected in up to 90% of cases) (Gennari et al., 2019). The disease was first described in England, in 1877, by Sir James Paget who defined it with the name of “osteitis deformans” (Paget, 1877). However, this morbid picture is certainly older, so much that the first pagetic femur dates back to about three thousand years ago (Ortner and Putschar, 1981). Indeed, albeit with limits, lesions somewhat suggestive of the disease have even been supposed in vertebral bodies of dinosaurs from the late Palaeozoic to the mid Mesozoic eras (Witzmann et al., 2011; Haridy et al., 2019).

The prevalence of PDB is difficult to quantify due to the fact that it is often asymptomatic, especially in the first years after onset. It is very rare in people under the age of 30 years and it is generally diagnosed after the age of 50. On the basis of initial autopsy findings reported in the 1930s, prevalence rates around 2%–4% have been estimated for subjects over the age of 50, and the probability of its occurrence increased with age reaching 10% or above in very elderly subjects (Cooper et al., 2006; Gennari et al., 2018). No more up-to-date data is available from autopsy studies. Moreover PDB appears to be prevalent in males (male to female ratio variable between 1.5 and 2) and was more frequently described in European Countries, with a particularly higher prevalence in UK (reaching 4%–6%), or in North America (up to 3% in subjects of European origin), Australia and New Zealand, where it mainly affects the descendants of the colonizers (Cooper et al., 2006; Gennari et al., 2018). Conversely it was more rarely described in Africa, Asia and the Scandinavian countries. However, information about PDB prevalence in non-Caucasian populations is very limited, and patient series of Asian and Indian ancestry have very recently been described (Asirvatham et al., 2020; Wang et al., 2020). There are also restricted geographic areas with a particularly high PDB prevalence such as the Lancashire region in England, the “La Cabrera” district in Spain and some districts of the Campania region in Italy, where an increased disease severity was also reported (Barker et al., 1980; Lopez-Abente et al., 2003; Gennari et al., 2006; Rendina et al., 2006). Most epidemiological studies have documented a gradual decrease in the prevalence and incidence of PDB over the years, which is associated with a parallel decline in mortality and clinical severity (Cundy et al., 1997; Cooper et al., 1999; Cook et al., 2021). More recent estimates in subjects aged 45 years and over suggested a PDB prevalence below 1%, including the high prevalence areas of United Kingdom (Abdulla et al., 2018; Mazières et al., 2018). In a last report from UK primary care records, the overall standardized incidence of clinically diagnosed PDB decreased from 0.75/10,000 person-years in 1999 to 0.20/10,000 person-years in 2015 (Cook et al., 2021).

The peculiar feature of PDB is represented by an exaggerated increase in bone resorption followed by a phase of increased formation; it is believed that this is due to a defect in the osteoclasts which are increased in number, size and quantity of nuclei per cell (Reddy et al., 1999; Roodman and Windle, 2005). Pagetic bone is the site of intense metabolic activity and is richly vascularized; however, despite the increase in size the pagetic tissue still has poor biomechanical efficiency due to its structural disorganization. These features can result in various clinical consequences, such as a tendency to compress the nerve structures within the affected bones, the presence of bone deformities, a greater risk of fracture, and a frequent association with forms of osteoarthritis secondary to skeletal deformity and abnormal joint load (Gennari et al., 2019). In some cases (less than 1%) neoplastic degeneration of pagetic tissue in osteosarcomas, fibrosarcomas, chondrosarcomas, or giant cell tumor (GCT) is also described (Hansen et al., 2006; Rendina et al., 2015).

**FIGURE 1:**
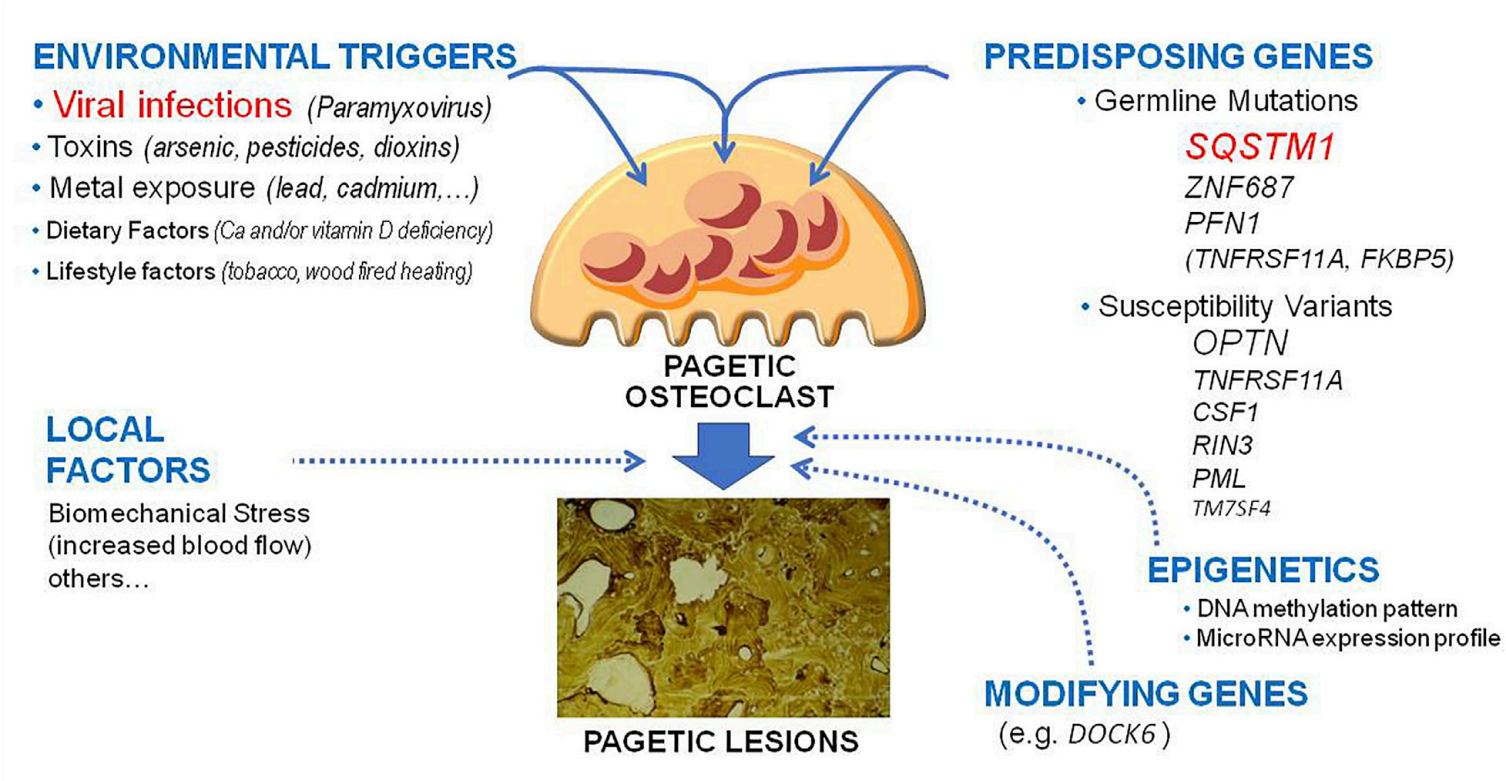
Updated pathogenetic model of Paget’s disease of bone. The figure underlines the different genetic, environmental lifestyle and epigenetic factors and their potential reciprocal interactions in the pathogenesis of pagetic lesions.

The cause of PDB remains in part unknown. Two main hypotheses have been originally proposed, the genetic one and the one linked to the presence of environmental triggers (Roodman and Windle, 2005; Ralston et al., 2008; Gennari et al., 2019). Over the past two decades, important advances have been made in the field of cellular and molecular biology of PDB, and some genetic mutations have been identified as a possible cause of the disorder in up to 20%–30% of cases (Gennari et al., 2019; Makaram and Ralston, 2021). However, at least in a subset of cases, genetic susceptibility might not be a sufficient condition for the clinical development of PDB without the concomitant intervention of other factors, such as those related to a viral infection (Figure 1).

## 2 Genetics of Paget’s disease of bone

The presence of a familial predisposition in PDB has been known for many years. Numerous epidemiological studies have in fact indicated a familial clustering in 15%–30% of patients, suggesting the possible role of genetic factors (Montagu, 1949; Siris et al., 1991), even though lower estimates have been also reported in some other studies (Eekhoff et al., 2004). Indeed, it is likely that the percentage of familial cases is underestimated, since the disease generally arises after 40 years of age and can remain asymptomatic for a long time. In this regard, an in-depth study in which detailed clinical research was carried out in first degree relatives of patients with the disease would have shown familial clustering in up to 40% of cases (Morales-Piga et al., 1995). It has also been estimated that the risk of PDB in first degree relatives of subjects suffering from the disease is about 7–10 times higher than in subjects without a family history (Siris et al., 1991), regardless of whether germline mutations of genes currently known related to PDB have been identified in affected subjects. The mode of inheritance appears to be autosomal dominant, although the existence of families with few affected members suggests incomplete penetrance and variable expression of the responsible gene or genes. A number of rare genetic disorders sharing some histopathological, radiological, and clinical overlap with PDB and even a similar genetic background have been also described (Ralston and Taylor, 2019). In the past two decades germline mutations in more than one gene and a number of predisposing common variants (i.e., polymorphisms) have been identified as the likely pathogenetic cause of PDB in a relevant number of patients (Table 1). Most of them affect the nuclear factor kappa-light-chain-enhancer of activated B cells (NFκB) signalling pathway and are implicated in the regulation of osteoclast differentiation and function, albeit at least three mutated genes are also involved in cell autophagy. Among the many functions attributed to NFκB, the one inherent osteoclastic pathophysiology is its modulating role in the differentiation of preosteoclasts into mature osteoclasts and their functional activation (Boyce et al., 2015).

**TABLE 1:** Germline Mutations associated with PDB and/or PDB related disorders and their functions.

Gene	Encoded product	Bone related functions	PDB and PDB-related disease
SQSTM1	P62/sequestosome1	regulation of autophagy and NFκB signaling	Familial PDB
TNFRSF11A	RANK	master regulator of osteoclast formation and survival	Early onset PDB (rarely)
VCP	Valosin containing protein	role in protein degradation and autophagy, intracellular membrane fusion, DNA repair and replication; regulation of the cell cycle; activation of the NF-kB pathway	Inclusion Body Myopathy, PDB, FTD” (IBMPFD, now renamed MSP1)
ZNF687	Zink Finger Protein 687	unknown (part of the transcriptional regulator complex Z3)	Severe, early onset, PDB with higher risk of neoplastic degeneration in GCT
FKBP5	FK506-binding protein 51 (FKBP51)	regulator of NF-κB activation and Akt phosphorylation; scaffolding protein and autophagy inducer; regulation of microtubule dynamics	Polyostotic PDB (described in 2 Chinese Han families)
PFN1	Profilin 1	negative regulator of the NF-kB signaling; also involved in the turnover and restructuring of the actin cytoskeleton	Severe, early onset, PDB with higher risk of neoplastic degeneration

### 2.1 Genetic mutations associated with Paget’s disease of bone

#### 2.1.1 Sequestosome 1 gene

Mutations affecting the sequestosome 1 gene (*SQSTM1*, within the PDB3 locus on chromosome 5q35 emerged from initial linkage analysis studies) currently represent the major genetic cause of adult PDB and have been described in approximately 25%–40% of familial cases and in up to 10%–15% sporadic cases (i.e., those patients without documented familial clustering) in various patient series (Hocking et al., 2002; Laurin et al., 2002; Johnson-Pais et al., 2003a; Eekhoff et al., 2004; Good et al., 2004; Hocking et al., 2004; Falchetti et al., 2009; Rea et al., 2009; Gennari et al., 2010). The most common of these mutations is represented by the proline-leucine amino acid substitution in codon 392 (P392L) at the level of exon 8 (Gennari et al., 2010). These *SQSTM1* mutations are generally heterozygous mutations, but rare cases of homozygosity have been described, with both alleles of the *SQSTM1* gene mutated (Rea et al., 2009). Following the two positional cloning studies in 2002 (Hocking et al., 2002; Laurin et al., 2002), at least 30 different mutations is *SQSTM1* have been associated with PDB. The majority of these mutations affect the terminal portion of the gene and in particular the ubiquitin-binding domain (UBA domain) of the protein. In most of the series analyzed to date, it has been shown that patients with the *SQSTM1* mutation generally have a more severe form of the disease than patients without the mutation, with an earlier onset and a greater number of bones involved (Falchetti et al., 2009; Rea et al., 2009; Gennari et al., 2010; Visconti et al., 2010; Chung et al., 2011). Furthermore, genotype-phenotype correlation have suggested that patients with mutations that cause a stop codon and therefore the lack of synthesis or the synthesis of a truncated form of the protein (missing most of the UBA domain), tend to have a more severe and extensive disease compared to patients with “missense” mutations causing the change in one or more amino acids (Gennari et al., 2010; Visconti et al., 2010). However, there is considerable heterogeneity in the clinical manifestations of the disease even in patients with the same mutation, and even within the same family (Laurin et al., 2002; Gennari et al., 2010). Moreover, in keeping with the secular trends, the clinical expressiveness seems to have decreased over time in *SQSTM1* mutation carriers, so that the phenotype of PDB is attenuated and less extensive in the more recent generations, and it is delayed by at least 10 years (Bolland et al., 2007; Cundy et al., 2015; Dessay et al., 2020). The reasons for the delayed penetrance of PDB in these offspring remain unknown, but could be likely justified by a reduction in exposure to the putative environmental trigger(s).

The *SQSTM1* gene encodes a protein called p62, which is ubiquitously expressed and contains many structural domains, including the SH2-binding domain (Src homology 2), the atypical-PKC-interacting domain (AID), a ZZ domain, the TRAF6 ligand (TNFR-associated factor 6) two PEST motifs and the UBA domain (Joung et al., 1996). These domains exert several functions in different signaling and ubiquitin binding pathways that are relevant for cell autophagy, survival, or response to inflammatory and oxidative stress but also regulate the transcriptional activation and protein recruitment to endosomes (Joung et al., 1996; Duran et al., 2004). It is also known that p62 acts as an adaptor protein in the NFκB signaling pathway, downstream of the receptor activator of NFκB (RANK, a signaling receptor that plays a relevant role in osteoclast differentiation and function), where it binds molecules such as RIP (receptor interacting with proteins) and TRAF6 to the aPKC or other factors such as CYLD (a deubiquinating factor exerting an inhibitory effect on RANKL signaling) (Layfield et al., 2006; Jin et al., 2008; Sundaram et al., 2011). Most of the know *SQSTM1* mutations associated with PDB have deleterious effects on ubiquitin binding by p62 *in vitro* (causing either reduced UBA domain stability or affecting the ubiquitin binding interface) and there is evidence of an inverse relationship between ubiquitin binding function or the ability of different mutations to activate NFκB signaling *in vitro* and disease severity (Layfield et al., 2006; Goode et al., 2014). Thus, it has been speculated that the loss of ubiquitin binding by the mutated p62 protein might affect its interaction with ubiquitylated osteoclast proteins, leading to overstimulation of NFκB pathway and enhanced osteoclast formation and activity (Duran et al., 2004; Rea et al., 2013). Consistent with this hypothesis, osteoclasts derived from peripheral blood monocytes of *SQSTM1* mutation carriers showed increased sensitivity to RANKL and high bone resorption capacity *in vitro* when compared with those derived from control monocytes (Chamoux et al., 2009; Rea et al., 2013). However, PDB-causing *SQSTM1* mutations located outside the UBA domain with little or no effects on ubiquitin binding have been described, and other domains of the p62 protein, such as AID, ZZ and the one binding TRAF6, also exert a relevant effect on osteoclasts (Goode and Layfield, 2010; Rea et al., 2013), suggesting more complex pathogenetic mechanisms in the presence of *SQSTM1* mutation. This might also explain the conflicting results emerged to date from studies in *SQSTM1* knockout (KO) mice or animal models with the p62 P394L mutation, corresponding to the P392L mutation observed in human (Duran et al., 2004; Hiruma et al., 2008; Daroszewska et al., 2011; Kurihara et al., 2011; Zach et al., 2018). In fact, while a first study in *SQSTM1* KO mice showed defective osteoclastogenesis due to impaired activation of Nfatc1 and NFκB, without a clear skeletal phenotype (Duran et al., 2004), a more recent and detailed analysis of an independently generated *SQSTM1* KO model reported enhanced activation of osteoclastogenesis and osteoclast activity (with a robust activation of Nfatc1 and NFκB), together with the development of PDB-like osteolytic lesions (Zach et al., 2018). Likewise, albeit there is general consensus about an increased sensitivity to RANKL in osteoclasts derived from the *SQSTM1* P394L mice model, there are still contrasting data about the development of PDB-like lesion in these mice (Hiruma et al., 2008; Daroszewska et al., 2011; Kurihara et al., 2011). A potential explanation for the divergent results might be related to the age differences between the studies and the different skeletal sites tested for the presence of pagetic lesions (vertebral vs. lower extremity bone). Very recently, a new interactor protein of p62, Ajuba, has been identified as an additional component of the SQSTM1/p62 protein complexes involved in NFκB signaling (Sultana et al., 2021). *In vitro*, Ajuba was shown to activate NFκB activity, while co-expression with SQSTM1/p62 inhibited this activation in an UBA domain dependent manner. Thus, either the lack of *SQSTM1* expression or the presence of p62 forms with inactive UBA domain (as occurs with typical, PDB-related *SQSTM1* mutations) might lead to increased or prolonged Ajuba-induced NFκB signaling and enhanced osteoclastogenesis.

While heritable mutations occurring in the germ cells (so called germline mutations) involve all nucleated cells deriving from them, somatic mutations may occur at any cell division from the first cleavage of the zygote to the lifelong cell divisions in an individual, and consequently affect all somatic cells descended from the original mutated cell. Interestingly, evidence of somatic *SQSTM1* mutations in sporadic PDB and pagetic osteosarcoma suggested a role for *SQSTM1* in both sporadic and inherited PDB (Merchant et al., 2009) and might in part explain the focal nature of the disorder. In particular, the *SQSTM1* (C1215T) mutation was reported at somatic level in samples from sporadic pagetic osteosarcoma patients, with the normal adjacent tissue from these tumors lacking this mutation, as expected for somatic mutational events. Equally interesting is the fact that *SQSTM1* mutations were not found in primary adolescent, non pagetic, osteosarcomas (Merchant et al., 2009). Conversely, a similar analysis in osteoblast and bone marrow cells cultures from 28 PDB patients did not identify somatic mutations (Matthews et al., 2009). About 6 years after these preliminary reports, another study identified SQSTM1/P392L mutation at post-zygotic level, restricted to the monocytic lineage, in about 5% of analyzed PDB patients and 1.5% of controls. Interestingly, PDB patients carrying such a mutation exhibited a bone phenotype milder than PDB patients with the same mutation but at germline level (Guay-Bélanger et al., 2015).

#### 2.1.2 TNFRSF11A gene

Mutations in the *TNFRSF11A* gene, which encodes for RANK, were the first to be associated with isolated PDB cases and other, so called, PDB-like disorders, such as familial expansile osteolysis (FEO) and expansile skeletal hyperphosphatasia (ESH) (Hughes et al., 2000). This gene is located within the chromosomal region 18q21.1-22, containing the PDB2 locus. The RANK protein is a member of the TNFR (tumor necrosis factor receptor) superfamily, strongly expressed on osteoclasts and their precursors and essential for osteoclastic differentiation and activity (Hofbauer and Heufelder, 2000). Mutational screening of the *TNFRSF11A* gene revealed different insertions at the level of exon 1, mutations that result in the duplication of amino acid sequences in the RANK signal peptide (Hughes et al., 2000). To date, different heterozygous in-frame tandem duplications of variable length, elongating the signal peptide of RANK, have been described, most of them associated with rare PDB-like disorders (Ralston and Taylor, 2019). The 84dup18 (duplication of 18 base pairs in position 84) and the closely related one, 83dup18, have been found in most of the cases of FEO described so far (Hughes et al., 2000; Palenzuela et al., 2002; Johnson-Pais et al., 2003b); the 84dup15 mutation was found families with ESH (Whyte and Hughes, 2002); the 90dup12 was associated with panostotic expansile bone disease (Schafer et al., 2014); while the 75dup27 mutation was described in a Japanese family with an early and severe form of PDB, associated with unexplained tooth loss and bony enlargement of the small joints of the hands (Nakatsuka et al., 2003; Merchant et al., 2009). Of interest, in these disorders, the severity of the associated skeletal disease appears inversely related to the duplication’s length. Moreover, a different duplication, 87dup15, has been more recently described in a 13-year-old Bolivian girl affected with the so called “Juvenile PDB” (also named familial idiopathic hyperphosphatasia) (Whyte et al., 2014), an autosomal recessive disorder manifesting extremely fast skeletal remodeling, which is usually caused by loss-of-function mutations within *TNFRSF11B* gene, encoding osteoprotegerin (OPG), the decoy receptor of RANK-ligand (Whyte et al., 2002). Consequently, the hypothesis that FEO, ESH, and the few cases of early-onset PDB associated with *TNFRSF11A* mutation might represent slightly different manifestations of a common condition (that is different from classical PDB) cannot be excluded.

Initial functional studies suggested that both 84dup18 and 75dup27 mutations cause a defective cleavage of the RANK signal peptide resulting in the activation of the NFκB signal cascade, although the exact mechanism of these processes was unclear (Merchant et al., 2009). More recently a mouse model carrying the 75dup27 mutation was generated, which prospects a different pathogenetic mechanism (Alonso et al., 2021). Consistent with the clinical reports, mice heterozygous for the mutation developed a PDB-like disease with focal osteolytic lesions in the hind limbs with increasing age. However, functional *in vitro* studies showed that RANK ligand-induced osteoclast formation and signaling was impaired, while osteoclast survival was increased independent of RANKL stimulation. Surprisingly, 75dup27 homozygous mice showed osteopetrosis at birth, with complete absence of osteoclasts and an impaired osteoclastogenesis in response to RANKL and macrophage colony-stimulating factor (M-CSF) stimulation.

To date, apart from two additional forms of early onset PDB and different 27bp duplications (78dup27 and 77dup27) described, respectively, in single kindreds of Chinese and Mexican descent (Ke et al., 2009; Iwamoto et al., 2020), no other cases of mutations of the *TNFRSF11A* gene have been described in both familial and sporadic cases of PDB, although some polymorphisms of this gene might be associated with the risk of developing the classic form of the disorder (see Section 2.2, below) (Chung et al., 2010).

#### 2.1.3 Valosin containing protein gene and other multisystem proteinopathy genes

Mutations in the VCP gene (encoding valosin containing protein), at the 9p21 locus, were identified as the cause of the autosomal dominant syndrome “Inclusion Body Myopathy, Paget’s disease of bone, Fronto-temporal Dementia” (IBMPFD), characterized by skeletal manifestations similar to classical PDB, associated with myopathy, amyotrophic lateral sclerosis (ALS) and fronto-temporal dementia (Kovach et al., 2001; Watts et al., 2004). Myopathy is the most frequent feature in IBMPFD families, occurring in up to 90% of individuals, while PDB and dementia have been described in 43% and 37% of cases, respectively (Ralston and Taylor, 2019). Albeit the clinical and radiologic features are similar to classical PDB (including the presence of asymptomatic cases), the skeletal manifestation often occur at an earlier age (around 40–45 years). Moreover, increased mortality has been reported in IBMPFD patients due to respiratory insufficiency and/or cardiac failure, with a life expectancy around 58 years (Ralston and Taylor, 2019).

Since its original description, IBMPFD has been reclassified and included among the group of “Multisystem Proteinopathies” (MSP), disorders with a broader phenotypic spectrum in which different neuromuscular disfunctions (also including parkinsonism and motor neuron disease) may be accompanied by pagetic lesions (Taylor, 2015). The *VCP* gene encodes for an enzymatic protein involved in cell division, fusion of membranes within cells, reassembling cell structures after cell division, prevention of cells apoptosis, and repair of the damaged DNA. In particular, this protein is also linked to the ubiquitin-proteasome system, and therefore, most likely, contributes to the regulation of a correct autophagy process. To date, more than 45 different pathogenic *VCP* mutations have now been identified in patients with IBMPFD (now classified as MSP1) (Ralston and Taylor, 2019). Most of them affect exons 3–5, encoding the N-terminal domain of the protein, which is responsible for binding to ubiquitin-complexed proteins, which will be degraded by the proteasome (Ralston and Taylor, 2019). Thus, likewise *SQSTM1* mutations, disease-causing mutations in *VCP* may impair the scaffold function required to transport proteins to the proteasome for degradation, causing them to accumulate in the cytoplasm as inclusion bodies which are typically observed in myocites and neurons. In osteoclasts this also determines a reduced degradation of proteins involved in the NFκB signaling pathway, with prolonged activation of this pathway and consequent cell hyperactivity and bone resorption (Ralston and Taylor, 2019). An increased avidity of VCP mutant proteins for the NFκB inhibitor IkB has been also described as an additional mechanism of increased NFκB activity. In order to better understand the pathogenesis of MSP disorders, transgenic mice models overexpressing known VCP mutations have been generated. Together with muscle weakness and behavioural abnormalities these mice showed an osteopenic phenotype and developed focal osteosclerotic lesions within the internal cavity of long bones (Custer et al., 2010).

Overall, VCP mutations are estimated to account for about 50% of cases of MSP (the so called MSP1 form) and other mutations have been described involving hnRNPA2B1, hnRNPA1, and MATR3 genes (Taylor, 2015; Ralston and Taylor, 2019). The mechanisms by which these mutations cause the pagetic degenerations observed in some affected members remain to be established. Of interest, mutations in *SQSTM1* were also rarely described in patients with neuromuscular degeneration and other typical clinical features of MSP, either in the presence or absence of PDB (Teyssou et al., 2013; Bucelli et al., 2015). Likewise, *SQSTM1* mutations have been also detected in a limited number of patients with only frontotemporal dementia or ALS (Fecto and Siddique, 2012; Le Ber et al., 2013). The reason for such a different clinical presentation, even in the presence of a same *SQSTM1* mutation, remains unknown but it is likely that other genetic mutations or susceptibility alleles might predispose *SQSTM1*-mutated patients to one ore more clinical manifestations described in MSP, including PDB. In keeping with this hypothesis a N357S genetic variant of *TIA1* gene (encoding for a key component of cytosolic stress granules) has been shown to interact with *SQSTM1* mutations to cause distal myopathy, whereas both *SQSTM1* mutation and the N357S genetic variant alone do not cause myopathy (Lee et al., 2018). Somatic mutations of the *VCP* gene have not been identified neither in pagetic tissue nor in bone tumors, although some human cancers exhibited them (Tate et al., 2019).

#### 2.1.4 ZNF687 gene

In 2016, a whole-exome sequencing analysis of a large Italian PDB-pedigree with 14 affected members (four of whom developed GCTs at multiple pagetic skeletal sites) identified a missense P937R mutation in a new gene, *ZNF687* (Divisato et al., 2016). The mutation co-segregated with the clinical phenotype in all affected family members of the pedigree and was also identified in 2 out of 615 Italian PDB patients, in 2 out of 339 PDB cases from a multiethnic North-American cohort, and in seven unrelated cases with giant cell tumor degeneration at pagetic bones (Divisato et al., 2016). Moreover, a different mutation of *ZNF687* (S242I) was also described in another PDB family (Divisato et al., 2016). In a subsequent analysis, the P937 mutation was also found in a 45-year-old black American woman with polyostotic PDB and in tumor tissue derived from 1 undifferentiated pagetic sarcoma, while a new *ZNF687* mutation (R331W) was identified in 1 of 28 pagetic osteosarcomas (Scotto di Carlo et al., 2020a). From the clinical point of view, PDB cases harboring *ZNF687* mutations generally have a severe form of PDB, with an earlier onset and greater number of affected skeletal sites than other PDB cases, including patients with *SQSTM1* mutation (Divisato et al., 2016). Of interest, remarkable clinical and molecular differences have been reported between pagetic and non pagetic GCTs (Rendina et al., 2015). In fact, GCT generally complicates a longstanding, active, and polyostotic PDB, involving only skeletal sites affected by the disease or, rarely, extra-skeletal tissues adjacent to them. In contrast with the prevalent involvement of axial the skeleton in adults with PDB, GCT unrelated to PDB preferentially occurs in Asian females aged below 40 years and mostly involves the epi‐metaphysis of long bones, in particular femurs and tibiae (Werner, 2006). In addition, pagetic GCT is frequently multifocal (in up to 25% of cases) and is associated with severe disease and reduced life expectancy, with a 5-years survival rate below 50% as compared to 96%–100% described in non-pagetic GCT (Rendina et al., 2015). These clinical differences might also be related to a different genetic background, since more than 90% of non pagetic GCT are associated with recurrent somatic mutations of the *H3F3A* gene, encoding the histone variant H3.3, that have rarely described in pagetic GCTs (Scotto di Carlo et al., 2020b). The increased prevalence of PDB‐GCT cases in families from Southern Italy (up to 50% of pagetic GCT cases described so far) also suggests a strong genetic basis with a founder effect.

The *ZNF687* gene encodes for a zinc finger protein that is part of the transcriptional regulator complex Z3 (Malovannaya et al., 2011), is highly expressed during osteoclastogenesis and is upregulated in pagetic GCTs (Divisato et al., 2016). The exact function of this gene in bone metabolism and the pathogenetic mechanism leading to the development of PDB in *ZNF687* mutation carriers and/or to neoplastic degeneration of pagetic tissue remain unknown.

#### 2.1.5 FKBP5 gene

In 2017 a missense mutation in *FKBP5* gene (V55L) was related to PDB in a single Chinese pedigree with four affected polyostotic cases (Lu et al., 2017). The gene encodes for FK506-binding protein 51 (FKBP51), a known regulator of NFκB activation and Akt phosphorylation (Bouwmeester et al., 2004; Lu et al., 2017). A *FKBP51* ^V55L^ knock-in transgenic mice model was also generated to better characterize the skeletal implications of the mutation. Of interest, osteoclast precursors derived from the transgenic mice showed enhanced Akt phosphorylation and RANKL sensitivity, while mature osteoclasts exhibited a more intensive bone resorbing capacity than osteoclasts derived from the control mice (Lu et al., 2017). In keeping with the *in vitro* data, osteolytic lesions resembling the initial phase of PDB were also evidenced by micro-CT in three-dimensional reconstruction of distal femurs of *FKBP51* ^V55L^ mice. However, at least in 10-months old animals, the focal increase in bone resorption was not followed by aberrant osteoblastic activity, osteosclerosis and increase in bone size, as typically observed in human PDB. To date the pathogenetic mechanism of *FKBP5* mutations in PDB is unknown and this finding remains restricted to a single PDB pedigree of Asian descent. Intriguingly, FKBP51 also acts as a scaffolding protein organizing protein complexes and functions as an autophagy inducer (Gassen et al., 2015; Fries et al., 2017), albeit no aberrant autophagy was described in the A *FKBP51* ^V55L^ mice during osteoclast differentiation *in vitro* (Lu et al., 2017). In addition, *FKBP51* is also involved in the regulation of the cytoskeleton, more specifically microtubule dynamics (Fries et al., 2017).

#### 2.1.6 PFN1 gene

Very recently, mutations in the *PFN1* gene were separately described in two Italian pedigrees affected with an early onset form of PDB (Scotto di Carlo et al., 2020c; Merlotti et al., 2020) and in two Chinese Han PDB families complicated by GCT (Wei et al., 2021). The same 4-nucleotide deletion giving rise to a frameshift mutation (D107Rfs*3) was found in the Italian families and in one out two Chinese families, as well as in a sporadic Italian PDB case without know family history for the disease. Novel *PFN1* mutations were described in the second Chinese family (L112P) and in a sporadic early-onset PDB patient of Asian ancestry (heterozygous 1-bp deletion c.324_324delG) with GCT. Remarkably, all patients had early onset, polyostotic PDB with skull involvement, which appears a typical characteristic of the disease caused by *PFN1* mutation, since hyperostosis and osteolytic lesions of the skull were also found in a 17 years old, asymptomatic, unaffected carrier of the D107Rfs*3 mutation (Merlotti et al., 2020). Other peculiar phenotype characteristics included a rather symmetrical and extensive skeletal involvement, the presence of severe, early onset, osteoarthritis at the spine and major joints (occurring between 25 and 50 years) and an increased prevalence of fractures or neoplastic degeneration in either osteosarcoma (described in 21% of Italian patients) or GCT (described in 31% of Chinese cases). Moreover, reduced response to aminobisphosphonates, including zoledronic acid, requiring multiple infusions to control bone pain and achieve biochemical remission over a long term was described in most of these cases (Merlotti et al., 2020; Wei et al., 2021). Osteoclasts derived from peripheral blood mononuclear cells of *PFN1* mutation carriers showed PDB-like features such as a larger size and a higher number of nuclei (Scotto di Carlo et al., 2020c; Merlotti et al., 2020; Wei et al., 2021).

The *PFN1* gene encodes for profilin 1, a highly conserved and ubiquitously expressed protein among vertebrates, and member of the profilin family of small actin-binding proteins involved in the turnover and restructuring of the actin cytoskeleton (Carlsson et al., 1977; Alkam et al., 2017), that might be relevant for osteoblast biology (Luxenburg et al., 2007; Han et al., 2019). In addition, profilin 1 was shown to suppress NFκB activity in the regulation of osteoclast differentiation and to prevent the degradation of the phosphatase PTEN, a known negative modulator of osteoclast differentiation (Zaidi and Manna, 2016). Of interest, previous experimental evidences already underlined the relevance of profilin 1 for bone biology, since osteoclast specific deletion of *PFN1* increased cell motility, podosome formation, size, and bone resorptive activity of osteoclasts *in vitro* and caused several bone abnormalities *in vivo*, including dwarfism, reduced trabecular bone mass and osteolytic lesions (Shirakawa et al., 2019). Moreover, as for other PDB genes (e.g., *SQSTM1* or *VCP*), *PFN1* mutations were previously described in patients with familial ALS (Wu et al., 2012).

After the discovery of *PFN1* mutations in PDB, a transgenic mice model with the truncating D107Rfs*3 mutation of *PFN1* gene was recently established (Wei et al., 2021). While the homozygous mutation was embrionically lethal (likewise in the *PFN1* KO mice), heterozygous transgenic mice were smaller in size, and showed accelerated osteoclast differentiation, with deformed craniofacial bones and the presence of focal PDB-like lesions (Wei et al., 2021). Reduced profilin 1 expression was also demonstrated in femur sections from transgenic mice using immunohistochemistry staining (Wei et al., 2021). Taken all together these experimental observations suggest that the *PFN1* mutations associated with PDB might confer a loss of function in profilin 1 activity and cause PDB-like features in the osteoclasts, likely due to enhanced cell motility, increased Nf-kB activity, and actin ring formation.

### 2.2 Other genetic pathways associated with Paget’s disease of bone: Genome wide association studies, single nucleotide polymorphisms association analysis, and modifying genes

Despite *SQSTM1* and other rarer mutations have been found in a relevant number of familial PDB-cases from different Countries, their prevalence remains very low in patients with sporadic disease, and at up to 40%–50% of familial cases do not yet have a recognized mutation. This suggests the presence of additional predisposition genes. Over the past years, molecular genetic studies in animal models of PDB, in cellular models of “pagetic” osteoclasts, and Genome Wide Association Studies (GWAS) in unrelated PDB individuals have contributed to identify other several potential genes and loci enabling the predisposition to develop PDB (Table 2).

**TABLE 2:** PDB-predisposing genetic variants (with chromosome location), reference SNP identification, encoded proteins, bone related functions and associated diseases different than PDB and/or PDB-related diseases.

Gene/chromosome region	References SNP (rs)	Encoded product	Methodological approach	Bone related functions	Diseases associated other than PDB or PDB-related diseases
OPTN gene/10p13	rs1561570	Optineurin	GWAS	regulation of autophagy and NFκB signaling	Glaucoma, Primary Open Angle and Amyotrophic Lateral Sclerosis 12 With or Without Frontotemporal Dementia (https://www.genecards.org/cgi-bin/carddisp.pl?gene=OPTN)
CSF1/1p13	rs484959	MCSF	GWAS	master regulator of osteoclast formation and survival	Pigmented Villonodular Synovitis and Tenosynovial Giant Cell Tumor (https://www.genecards.org/cgi-bin/carddisp.pl?gene=CSF1)
TNFRSF11A/18q21	rs3018362	RANK	GWAS	master regulator of osteoclast formation and survival	Osteopetrosis, Autosomal Recessive 7 (https://www.genecards.org/cgi-bin/carddisp.pl?gene=TNFRSF11A)
TNFRSF11A/18q21	rs1805034 (*V192A*)	RANK	Whole exome scanning	greater activation of NFκB signaling *in vitro* and with increased disease severity *in vivo*	Osteopetrosis, Autosomal Recessive 7 (https://www.genecards.org/cgi-bin/carddisp.pl?gene=TNFRSF11A)
PML/15q24	rs5742915	Phosphoprotein member of TRIM	GWAS	differentiation, survival and resorptive activity of osteoclasts (mice)	Acute Promyelocytic Leukemia and Rabies (https://www.genecards.org/cgi-bin/carddisp.pl?gene=PML)
RIN3/14q32	rs10498635	Ras and Rab 3 interactor protein	GWAS/fine mapping analysis	vesicular trafficking, expressed particularly in osteoclasts	Ciliary Dyskinesia, Primary, 6 (https://www.genecards.org/cgi-bin/carddisp.pl?gene=RIN3)
	rs117068593				
NUP205/7q33	rs4294134	Nucleoporin 205	GWAS	unknown	Nephrotic Syndrome, Type 13 and Genetic Steroid-Resistant Nephrotic Syndrome (https://www.genecards.org/cgi-bin/carddisp.pl?gene=NUP205)
TM7SF4/8q22	rs2458413	DC-STAMP	GWAS	fusion of osteoclast precursors to form multinucleated mature osteoclasts	Osteopetrosis, Autosomal Recessive 8 (https://www.genecards.org/cgi-bin/carddisp.pl?gene=DCSTAMP)
DOCK6/19p13.2 (p.Val45Ile)	—	Dedicator Of CytoKinesis (DOCK) family of atypical guanine nucleotide exchange factors	Whole exome scanning	role in actin cytoskeletal reorganization by activating the Rho GTPases Cdc42 and Rac1	Adams-Oliver Syndrome 2 and Adams-Oliver Syndrome (https://www.genecards.org/cgi-bin/carddisp.pl?gene=DOCK6)
				p.Val45Ile variant may decrease SRF-TF-like activity	
VCP/9p13.3	rs565070	Valosin Containing Protein	SNPs association study	role in protein degradation, intracellular membrane fusion, DNA repair and replication, regulation of the cell cycle, and activation of the NF-kappa B pathway	Frontotemporal Dementia and/o (https://www.genecards.org/cgi-bin/carddisp.pl?gene=VCP r Amyotrophic Lateral Sclerosis 6)

Thus, in 2010 and 2011 a GWAS approach was applied to large and multiethnic populations of PDB in order to identify novel genetic variants predisposing to the disease (Albagha et al., 2010; Albagha et al., 2011). Overall, the following candidate regions were identified, accounting for about 13% of the familial risk of PDB in *SQSTM1* negative patients: 1) 10p13 (rs1561570, within *OPTN* gene); 2) 1p13 (rs484959, near the *CSF1* gene); 3) 18q21 (rs3018362, near *TNFRSF11A* gene); 4) 15q24 (rs5742915, within *PML* gene); 5) 14q32 (rs10498635 within *RIN3* gene); 6) 7q33 (rs4294134, within*NUP205* gene); and 7) 8q22 (rs2458413, within *TM7SF4* gene). Remarkably, some of these regions such as *TNFRSF11A* (encoding for RANK, as described in Section 2.1.2), *CSF1* (encoding for macrophage colony-stimulating factor, MCSF), and *TM7SF4* (encoding for the dendritic cell-specific transmembrane protein, DC-STAMP) have a well-recognized role in regulating osteoclast formation and activity, making their pathogenic effect on PDB biologically plausible. In fact, while MCSF and RANKL are the key regulators of osteoclast formation and activity (Boyce et al., 2015), DC-STAMP is necessary for the fusion of osteoclast precursors into multinucleated, mature osteoclasts (Kukita et al., 2004; Yagi et al., 2005). The implication of other genes such as *OPTN*, *RIN3*, and *PML*, in bone physiology has been more recently suggested.

The *OPTN* gene encodes for optineurin, a protein that, likewise p62/SQSTM1, is involved in the regulation of autophagy and NFκB signaling (Zhu et al., 2007). A first study indicated that mice harboring a loss of function mutation in the ubiquitin-binding domain of *Optn* (*OptnD477N/D477N*) have enhanced bone turnover and that in osteoclast precursors optineurin acts as a negative regulator of RANK-induced NFκB activation (Obaid et al., 2015). Thus, since the risk rs1561570 allelic variant of *OPTN* was associated with reduced optineurin expression (Obaid et al., 2015), a plausible pathogenetic mechanism could be represented by an increase in NFκB activity and osteoclast formation and activity. However, only a limited number of these mice (10%) developed PDB like lesions (Obaid et al., 2015), making difficult the understanding of mechanistic process underlying the OPTN-PDB axis. Then, other animal studies using *Optn* KO model evidenced a recapitulation of the key clinical features observed in PDB patients, such as polyostotic osteolytic lesions, mixed-phase lesions, and increased bone turnover, and affecting all the studied animals between 16 and 22 months of age (Wong et al., 2020). Interestingly, when the authors investigated the *ex vivo* differentiation of primary osteoclasts noted that the absence of *Optn* caused an increased osteoclastogenesis. Moreover, *Optn*-deficient osteoclasts displayed a significantly decreased type I interferon (IFN) signature, due to both defective production of IFNβ and impaired signaling via the IFNα/βR, acting as a negative feedback loop for osteoclastogenesis and survival. Such data lead to the hypothesis that optineurin may have dual roles in the type-I IFN response to restrain osteoclast activation and bone resorption, thus representing a potential novel therapeutic target for PDB. Very recently, it has been also suggested that OPTN interacts with nuclear factor erythroid-derived factor 2-related factor 2 (NRF2), acting as a master regulator of the antioxidant response, hypothesizing a pathway through which RANKL-induced reactive oxygen species (ROS) might be relevant for osteoclastogenesis. A study on monocytes from mice knock out for *Optn* (*Optn−/−*) compared with the wild-type (*Optn+/+*) counterpart revealed that *OPTN* deficiency decreased the basal expression of NRF2, inhibited the expression of NRF2-responsive antioxidants, and increased basal and RANKL-induced intracellular ROS levels, thus leading to enhanced osteoclastogenesis. Such findings contribute to sustain a novel OPTN-mediated molecular mechanism to regulate the NRF2-mediated antioxidant response in osteoclasts, thus extending the therapeutic potential of optineurin in the aging process resulting from ROS-triggered oxidative stress, known to be also associated with PDB (Xue et al., 2021). While germline *OPTN* mutations were previously described in patients with primary open-angle glaucoma and ALS (Toth and Atkin, 2018), they have never been detected in PDB patients.

The *PML* gene encodes for a phosphoprotein member of the tripartite motif (TRIM) family. Such a protein localizes to nuclear bodies where it acts both as a transcription factor and tumor suppressor. It exhibits a cell-cycle related expression, and it enables the regulation of p53 response to oncogenic signals. *PML* is often involved in the translocation with the retinoic acid receptor alpha gene associated with acute promyelocytic leukemia (Bernardi and Pandolfi, 2007). Recently, it has been reported that the PDB-risk allele of rs5742915, located within the *PML* gene by the GWAS, associates with a lower PML expression and that PML expression in blood cells from PDB patients is lower than in non pagetic controls (Wani et al., 2022). In a mice model of *Pml* KO, the differentiation, survival and resorptive activity of osteoclasts was increased compared to wild type animals. Moreover, in KO animals, the inhibitory effect of IFN-γ on osteoclast formation resulted to be significantly blunted when compared to wild type mice, while the bone nodule formation was increased. Bone histomorphometry analysis revealed that *Pml* KO mice had also a high bone turnover with a parallel increase in bone resorption and mineral apposition rate indices, whereas micro-CT analysis of trabecular bone showed no differences with respect to the wild type animals. Thus, such findings strongly suggest that reduced expression of *PML* may predispose to PDB, identifying this protein as a novel regulator of bone metabolism (Wani et al., 2022).

Although, the exact function of *RIN3* in bone metabolism and the pathogenesis of PDB remains unknown, this gene encodes for the Ras and Rab three interactor protein which is involved in vesicular trafficking and is expressed in bone, particularly in osteoclasts (Vallet et al., 2015; Shen et al., 2022). Albeit *RIN3* mutations have not been detected so far in PDB patients, a fine mapping analysis within the *RIN3* locus on chromosome 14q32 identified some rare missense variants predicted to be highly pathogenic and resulting remarkably more prevalent in PDB cases than in controls (Vallet et al., 2015). Particularly, the rs10498635C allele on chromosome 14q32.12 was significantly associated with PDB in several European populations. Eighteen PDB-associated variants in *RIN3* locus, including 16 missense variants were studied by targeted sequencing of the 60 kb region in the *RIN3* gene in 741 PDB patients and 2,699 healthy controls. Three missense variants, the rs117068593T allele, the rs117068593C allele and the rs10498635C allele, were found to be most common in PDB than controls (Vallet et al., 2015). However, this study identified other several rare variants being more common in PDB cases than healthy controls, and although individually they did not reach statistically significant results, their combination did. Lately, 22 distinct variants were identified in a Belgian population of unrelated pagetic and healthy subjects. Eight of these variants were newly recognized missense variants, and 2 were in the 5’ untranslated region (De Ridder et al., 2019). Even though their results substantially confirmed the findings of the British cohort, the Belgian study strongly suggested a potentially modifying effect of the rs117068593, p. R279C variant on the age of onset of the disease, confirming *RIN3* as a potential gene modifier of the age of onset of the PDB. Moreover, albeit some of the variants described above affect the functional domains of the RIN3 protein, most of the variants associated with PDB are located in the noncoding regions of the RIN3 gene, likely altering its expression (Shen et al., 2022). While the mechanism by which these variants interact in the pathogenesis of PDB remains unknown, it has been shown that the targeted inactivation of the mouse *Rin3* gene leads to a reduction in osteoclast number and interferes with osteoclast activity, thus reducing bone resorption and leading to increased bone mass (Vallet et al., 2021). Thus, it is likely that PDB-predisposing variants of RIN3 in humans probably acts through a gain of function mechanism.

Despite the well recognized role of DC-STAMP in osteoclast biology, there is still limited and contrasting information about the mechanism through which the rs2458413 variant of its encoding gene (*TM7SF4*) identified in GWAS or other rare variants affect osteoclast phenotype and cause PDB (Laurier et al., 2017; Mullin et al., 2019; Sultana et al., 2019).

Importantly, an interaction between the novel variants identified by the GWAS or other polymorphic variants in the same candidate regions and *SQSTM1* mutations on the pagetic phenotype was described in different reports. In a first study assessing the effects of a *T575C* polymorphic variant of *TNFRSF11A* (rs1805034, which results in a *V192A* substitution in the RANK protein), the presence of the *C* allele (*A192*) was shown to synergistically interact with *SQSTM1* mutations on the PDB phenotype, leading to greater activation of NFκB signaling *in vitro* and with increased disease severity in PDB patients (Gianfrancesco et al., 2012). A subsequent, more detailed study investigated the clinical effects of all the seven allelic variants identified by the GWAS in a large multiethnic sample of 1940 PDB patients (Albagha et al., 2013). A cumulative risk allele score was specifically constructed by adding the GWAS variants together and relating this to disease severity, alone or in combination with *SQSTM1* mutations. In *SQSTM1*-negative patients, risk allele scores in the highest tertiles (thus bearing most of the GWAS variants related to PDB) were associated with enhanced disease severity compared with the lowest tertile, either in terms of number of affected sites or in disease severity score. Importantly, the risk allele score remained a significant predictor of disease severity when *SQSTM1-*positive individuals were considered. Likewise, in 2011, a study on 196 Belgian cases with sporadic PDB searched for possible correlation between single nucleotide polymorphisms (SNPs) of candidate genes, *TNFSF11A*, *VCP*, and *IL-6*, and disease development susceptibility (Chung et al., 2011). The latter gene was selected since IL-6 is known to be a stimulator of osteoclast formation, possibly acting as an autocrine/paracrine factor to enhance osteoclastogenesis in PDB patients (Kurihara et al., 1990; de la Mata et al., 1995). A total of 20 SNPs were used, nine for *TNFSF11A*, three for *VCP*, and eight for *IL-6*, respectively. Authors concluded that SNPs in *TNFSF11A* or *IL-6* were very unlikely to play a role in the of sporadic PDB in their study population. Interestingly, one SNP of *VCP* gene, referred to as rs565070, showed association with PDB, even if, as suggested by Authors themselves, replication of this finding in other populations is needed before it can be included in the list of PDB-associated genes (Chung et al., 2011).

Always remaining in the context of modifying genes, very recently Dessay et al. (2022) elegantly identified, by whole exome sequencing in two large unrelated French Canadian PDB families with germline P392L *SQSTM1* mutation, a clinically attenuating effect on PDB severity exerted by a V45I rare variant in the *DOCK6* gene. Remarkably, albeit both variants were separately found in PDB patients and gave rise to a pagetic phenotype of osteoclasts *in vitro* versus healthy controls, the *DOCK6* variant was found to delay the onset of PDB and attenuate the severity of osteoclast phenotype of PDB caused by the P392L mutation of *SQSTM1*, when both variants were present. DOCK6 protein belongs to the dedicator of cytokinesis (DOCK) family of atypical guanine nucleotide exchange factors, components of intracellular signaling networks and by interacting with small GTPases they probably play a role in actin cytoskeletal reorganization by activating the Rho GTPases Cdc42 and Rac1 (Miyamoto et al., 2007). The same Authors performed a structural bioinformatics analyses showing that the *SQSTM1* P392L mutation may decrease its possible intramolecular interaction with the serum response factor–transcription factor (SRF-TF)-like domain, while at the same time the V45I variant of *DOCK6* gene may decrease SRF-TF-like activity (Dessay et al., 2022). Indeed, homozygous or dominant negative heterozygous mutations of DOCK6 gene account for the Adams-Oliver syndrome-2, an autosomal recessive multiple congenital anomaly syndrome featured by aplasia cutis congenita and terminal transverse limb defects, in association with variable involvement of the brain, eyes, and cardiovascular system (Shaheen et al., 2011).

## 3 Environmental factors

Clinical and experimental evidences support the hypotheses that one or more environmental factors are necessary for the complete clinical expression of PDB. Indeed, the incomplete penetrance of disease in families with a documented genetic predisposition, the clinical observation that PDB does not affect the entire skeleton but involves only one or more skeletal sites, as well as the declining trends in the PDB incidence and severity observed over the years, strongly support the hypothesis that non-genetic factors are involved in the disease etiopathogenesis (Cooper et al., 2006; Singer, 2015; Gennari et al., 2019). Even though this assumption is generally accepted, the exact nature of these factors and whether or how they might interact with the genetic factors in the pathogenesis of PDB is poorly understood.

### 3.1 Viral factors

The possible involvement of viral factors in the pathogenesis of PDB has been reported since the early 1970s (Mills and Singer, 1976; Rebel et al., 1981), when virus-like inclusions were demonstrated first in the nucleus and then in the cytoplasm of pagetic osteoclasts using electron microscopy. These inclusions consist of groups of microtubules, which are present either in a compact paracrystalline array or are scattered in a more random fashion (Singer, 2015). The pagetic microtubules are similar to the nucleocapsids of two paramyxoviruses, measles virus (MV) and respiratory syncytial virus (RSV), showed identical dimension, and have been found in several studies in the past years (Mills et al., 1984; Basle et al., 1985; Basle et al., 1986; Mills et al., 1994; Reddy et al., 1995). More recently, Friedrichs et al. (2002) also identified the full-length sequence for the MV nucleocapsid gene in bone marrow obtained from a skeletal site showing the PDB pathognomonic changes from a PDB patient and more than 700 base pairs (bps) of MV sequence in three other PDB patients. These sequences were undetectable in four normal marrow samples studied simultaneously. Similar inclusions have been described in osteoclasts from patients with pycnodysostosis, osteopetrosis, and in foreign body giant cells of cases with primary oxalosis (Mills et al., 1988; Bianco et al., 1992). Other research groups have hypothesized an association between another paramyxovirus, the canine distemper virus (CDV), and PDB. The CDV is generally transmitted from infected dogs to humans through a bite or scratch and, indeed, several reports indicated that dog ownership was significantly more common in the PDB patients than in the control subjects (Anderson and O’Driscoll, 1986; O'Driscoll et al., 1990; Khan et al., 1996). Thus, Gordon et al. (1991) first demonstrated the presence of CDV mRNA in 11 of 25 bone biopsies from PDB patients using *in situ* hybridization analysis. These results were subsequently confirmed by Mee et al. (1998) that found the presence of CDV in all the tested samples from 15 PDB patients, using the *in situ* reverse transcriptase-polymerase chain reaction (IS-RT-PCR). Furthermore, *in vitro* reports suggested that CDV may induce osteoclastogenesis in human osteoclast precursors by activation of nuclear NFκB and SQSTM1/p62 (Selby et al., 2006), and more recently fusion and hemagglutinin proteins of CDV were shown to promote osteoclast formation through NFκB dependent and independent mechanisms (Wang et al., 2019). Altogether these results suggest that paramyxoviruses could induce in osteoclasts morphological changes resembling those described in PDB patients. However, many other attempts were made to replicate these findings in bone and blood samples from PDB patients, with negative results (Ralston et al., 1991; Birch et al., 1994; Helfrich et al., 2000; Ooi et al., 2000; Ralston et al., 2007; Matthews et al., 2008). While a different ability to detect the viral inclusions according to the technique used (from immunohistochemistry to *in situ* hybridization or RT-PCR) might in part explain such discordant findings (Hoyland et al., 2003), it has been also speculated that these inclusions might indeed originate from cell aggregates of undegraded proteins resulting from the dysregulation of cell autophagy consequent to an abnormal p62/SQSTM1 function (Vallet and Ralston, 2016a). However, the MV may persist for long time in cell types different form osteoclasts and osteoclast precursors and promote the disease in patients at a later time point. In this regard, Reddy et al. (1996) demonstrated that, in PDB patients, the MV nucleocapsid transcript expression is not restricted only to the osteoclast lineage but that also immature multipotent hematopoietic cells leading to granulocyte, erythrocyte, macrophage, and platelet expressed these transcripts. These results suggest that the pluripotent hematopoietic stem cells could be the initial target for MV infection in PDB patients.

In the last 20 years, animal models have been used to evaluate whether MV infection can induce the development of pagetic-like osteoclast and bone lesions. The MV genome consists of six genes encoding for the nucleocapsid, the matrix, the fusion, and hemagglutinin proteins, as well as the proteins L and P which both constitute the viral polymerase. Kurihara et al. (2000) first demonstrated that the transfection of normal human osteoclast precursors with the MV nucleocapsid protein (MVNP) caused the development of osteoclasts showing several functional and morphological characteristics of PDB osteoclasts. These characteristics include the increased cells size, the increased number of nuclei per cell, the increased capacity of bone resorption, the hypersensitivity to 1,25-(OH)_2_D_3_, and the increased TAF II-17 expression (Table 3). All these features were also described in osteoclasts developed *in vitro* from marrow samples of pagetic patients and were not observed when normal human osteoclast precursors were transfected with the MV matrix and fusion genes (Kurihara et al., 2000). Additional studies were performed using transgenic mice in which the human MV receptor, CD46, has been transfected in preosteoclastic cells. In effect, normal mice do not express the CD46 and are therefore resistant to MV infection. In CD46 transgenic mice the MV infection caused the development of osteoclasts showing functional and morphological properties similar both to those expressed by PDB osteoclasts and to those observed in normal human osteoclast’ precursors transfected *in vitro* (Reddy et al., 2001). Then, similar evidences were provided by the development of a transgenic mice model expressing the MVNP in the osteoclast lineage (TRAP-MVNP-mice). These mice not only had osteoclasts sharing all the phenotype characteristics of pagetic osteoclasts, but up to 30%–40% of them developed localized bone lesions in the L1-L4 vertebrae resembling those seen in patients with PDB (Kurihara et al., 2006; Miyagawa et al., 2020). Remarkably, later experiments in the same mice model demonstrated that, likewise observed in osteoclast from PDB patients (Roodman et al., 1992), MVNP expression induces the production of high levels of IL-6 from the osteoclast, which in turn increases expression of osteoclast IGF-1 in an autocrine manner (Teramachi et al., 2016). This increase in IGF-1 then upregulate the expression of coupling factors such as ephrinB2 in osteoclasts and EphB4 in osteoblasts (Teramachi et al., 2016; Miyagawa et al., 2020), leading to a parallel increase in bone formation that is a hallmark of PDB. Not only conditional deletion of IGF-1 in osteoclast of TRAP-MVNP-mice (MVNP/Igf1-cKO) totally blocked the increase in bone formation and the development of pagetic lesions, but, of interest mice harboring the knockin P394L mutation of *SQSTM1*, only exhibited increased bone resorption, but not formation (Teramachi et al., 2016; Miyagawa et al., 2020). In a different set of studies, the same Authors bred the TRAP-MVNP-mice to the knockin P394L mice to generate a p62KI/MVNP mice model, in order to assess the potential interaction between genetic and viral factors (Kurihara et al., 2011). While osteoclast precursors from p62KI/MVNP and TRAP-MVNP mice models showed all the typical features of pagetic osteoclast precursors, an incomplete phenotype was observed in osteoclasts from the knockin P394L animals (mainly characterized by the increased RANKL sensitivity of osteoclast precursors). Moreover, p62KI/MVNP mice generated the highest number of hyper-multinucleated PDB-like osteoclasts and up to 40% of them, between 18 and 26 months of age, developed focal bone lesions within vertebral bone, that completely replicated those seen in human PDB, with the presence of thickened trabeculae, woven bone and extensive marrow fibrosis (Kurihara et al., 2011). It was concluded that, at least in mice, *SQSTM1* mutation was mainly able to increase RANKL sensitivity of osteoclast precursors, whereas MVNP was responsible for most of the PDB-like features (Kurihara et al., 2011), including the increased IL-6 production that is necessary for the coupling of osteoclast and osteoblast activity, leading the parallel increase in bone formation.

**TABLE 3:** Major *in vitro* characteristics of pagetic osteoclasts from PDB patients, *SQSTM1* P394L mice or transgenic MVNP mice models.

Phenotype characteristic	Human pagetic osteoclasts	SQSTM1 P394L osteoclasts	Transgenic MVNP osteoclasts
Increased size	+	+	+
Increased number of nuclei	+	+	+
Increased sensitivity to RANKL	+	+	+
Increased sensitivity to 1,25(OH)2 vitamin D	+	−	+
Increased IL-6 production	+/−^a^	−	+

a Described by some but not all studies.

Other viruses, such as the swine influenza virus and the pig vesicular disease virus might also be implicated in the pathogenesis of PDB. These viruses are very difficult to identify and can persist in the asymptomatic phase for a long time in infected carriers (Marschall et al., 1996; Lin et al., 1998). In this regard, other epidemiological studies carried out in Italy and Spain have shown a higher prevalence of the disease in rural areas, especially in subjects in close contact with dogs and other animal species such as cattle and pigs (Lopez-Abente et al., 1997; Merlotti et al., 2005). It is therefore plausible that different viral infections, carried by different animal species, may be involved as possible triggers in the etiopathogenesis of PDB.

### 3.2 Other environmental risk factors

Environmental toxins such as arsenic or lead have been suggested as possible etiological agents of PDB. In 1974, a survey performed in the United Kingdom identified a cluster of six Lancashire towns where the average age-standardized prevalence of PDB was 6.3% compared with 4.3% in the remaining 25 and pointed out a possible link with the cotton industry (Barker et al., 1980). Considering that the two towns of the six with highest PDB prevalence are situated on estuaries whereas the two towns of the six with lowest PDB prevalence are above sea level, Barker and colleagues suggested a waterborne agent, the calcium arsenate [Ca_3_(AsO_4_)_2_], as the possible causative PDB agent. Indeed, Ca_3_(AsO_4_)_2_ was a pesticide largely used to protect the cotton crops against the boll weevils (*Anthonomus grandis*) until 1945, when it was progressively replaced by the less toxic Dichlorodiphenyltrichloroethane (DDT). Since the use of Ca_3_(AsO_4_)_2_ as pesticide and the PDB incidence in Lancashire significantly both diminished in parallel in the last decades of 20th century, it has been hypothesized a pathogenic link between Ca_3_(AsO_4_)_2_ use as pesticide and PDB (Lever, 2002). However, no direct evidence supports this hypothesis (Singer, 2015). On the other hand, Spencer et al. (1992) demonstrated a considerable occupational and environmental exposure to lead in 54 PDB patients. The lead amount was then determined in the bone specimen from PDB patients in two studies. In the first study, Barker et al. (1982) measured the lead levels in autopsy bone specimens form subject affected or unaffected by PDB and found that lead levels were lower in bone macroscopically affected by PDB compared to either normal bone from control individuals or bone from pagetic patients macroscopically unaffected by the disease. In the second study, Adachi et al. (1998), using bone biopsy samples, demonstrated that lead content in cortical bone was higher in PDB patients compared to patients with osteoporosis or with renal osteodystrophy. More recently, significant lower levels of some heavy metals, including arsenic and lead, were detected in urinary samples from PDB patients compared to healthy controls. Also, urinary cesium levels were lower in PDB compared to controls (Numan et al., 2019). However, in cellular models, an inhibitory effect of arsenic, lead, and cesium on osteoclast formation, on mean number of nuclei per cell, and on bone resorption were observed (Numan et al., 2019). An interaction with *SQSTM1* mutation was also described. In fact, in presence of cadmium, *SQSTM1* gene expression was upregulated in osteoclasts from patients with PDB versus healthy controls, particularly in osteoclasts from carriers of the *SQSTM1* mutation (Numan et al., 2019). Taken together, all the above results do not allow to reach a definitive conclusion about the possible involvement of heavy metals in the PDB pathogenesis.

Environmental factors other than metals have been also proposed as potential PDB triggers. Analysing self-reported medical questionnaires compiled by 864 pagetic patients and 500 healthy controls, all enrolled between spouses and friends of PDB patients, Siris observed that dietary calcium intake during childhood and adolescence (estimated by number of milk glasses consumed each day) was significantly lower in PDB patients compared to controls, suggesting a potential link between the low milk and calcium intake in childhood and adolescence and the susceptibility to PDB (Siris, 1994). Analysing the hospital discharges collected in England, Wales, and Scotland from 1966 to 1972, Barker and Gardner (1974) proposed a significant association between the occurrence of vitamin D deficiency related rickets in childhood and the development of PDB in elderly age. Despite this hypothesis is very intriguing considering the pleiotropic biological activities of vitamin D, evidences for an association between PDB and vitamin D deprivation in childhood are inconclusive at the present time. Likewise, excessive biomechanical loading has been proposed as potential trigger for PDB development (Solomon, 1979). This should also explain the focal nature of the disease. More recently, Michou et al. (2012), analysing all members of 18 French-Canadian pagetic families, established a significant association between current or past tobacco exposure and PDB occurrence. In fact, 43% of PDB patients were current or past smokers versus 18% of relatives unaffected by PDB. The same study group demonstrated that, in *in vitro* models, likewise observed for cadmium, *SQSTM1* expression was upregulated by tobacco smoke condensates, particularly in the presence of *SQSTM1* mutation (Numan et al., 2019). Using data from a French-Canadian cohort, Audet et al. (2017) demonstrated a significant association between wood fired heating in childhood and/or adolescence and both familial and no-familial form of PDB. In addition, familial form of PDB was significantly associated with residency near a mine and hunting. However, despite all these environmental factors have been associated to an increased risk of PBD in some patient cohorts, no conclusive evidence can be drawn.

## 4 MicroRNAs and epigenetics

As emerged above about the genetics of PDB, an incomplete penetrance of PDB has been reported in familial PDB, thus limiting the role of *SQSTM1* mutational analysis to the clinical practice (Falchetti et al., 2010). Moreover, the genetic heterogeneity of PDB and⁄or the presence of still unknown modifier genes or triggers able to control its clinical expression in subjects with germline mutations (e.g., in *SQSTM1* gene, as also in other of the above-mentioned genes) complicate our understanding of the genetic mechanisms of the disorder. Indeed, as previously described, exposure to several environmental triggers has been postulated, which could act as disease modifying agents affecting the susceptibility to develop PDB, especially in subjects who may be in some way genetically predisposed. In this respect, a potential unifying mechanism linking genetic and environmental factor in the pathogenesis of PDB, yet to be demonstrated as such, could be represented by epigenetics. Specifically, epigenetics deals with changes in gene expression not resulting directly from mutations of genomic DNA but possibly leading to inherited traits both at intra-generational and inter-generational level. In example, it was shown that DNA methylation can modulate the expression of *POMC* gene, encoding for the pro-opiomelanocortin molecule, thus representing a biological link between early childhood exposures and subsequent obese phenotype development (Candler et al., 2019).

Among the more investigated epigenetic mechanisms are DNA methylation, and histone modifications, both mechanisms capable of influencing the ability of one or more genes to be transcribed adequately or completely. Even bone tissue and bone cells may undergo these problems deriving from exposure to food and environmental factors (especially when prolonged over time) capable of negatively modulating the correct expression of relevant genes, for example, in the regulation of osteoclast differentiation, maturation and metabolic activation. Regarding PDB, a combined genomic analysis and machine learning approach identified several chemical signatures, which appeared remarkably different in the DNA of PDB patients (Diboun et al., 2021). In this study, a complex and technologically refined genome-wide profiling of DNA methylation at CpG islands was performed in a cohort of 253 PDB patients also including subject carriers of germline *SQSTM1* mutation, and 280 controls, in order to detect a predictive role of epigenetic markers to differentiate pagetic patients from non-pagetic controls. Combining genomic analysis and machine learning revealed 14 genome-wide significant differentially methylated sites between PDB patients and controls. Interestingly, most of these CpGs resides within/near genes with known to be functionally relevant in the pathogenesis of PDB, including osteoclast differentiation, or related to environmental triggers associated with PDB such as viral infection and mechanical loading. This further suggested the hypothesis that PDB pathogenesis could partly depend by environmental factors.

Likewise, microRNAs are small, non-coding, single-stranded RNAs that post-transcriptionally regulate gene expression, with an essential role in vertebrate development and different biological processes. Of interest a rising number of experimental reports suggested that miRNAs are involved in every step of osteogenesis and bone metabolism, by regulating the growth, differentiation, and activity of different cell systems inside and outside the skeleton, including osteoclast formation and activity (Gennari et al., 2017). Very recently, two small preliminary studies compared the miRNA expression profile of human osteoclasts derived form peripheral blood mononuclear cells of PDB patients and controls (Stephens et al., 2020; Nguyen et al., 2021). Some miRNAs appeared differently expressed between patients and controls. Target genes and enriched pathway analysis identified possible interactions between these miRNAs and apoptotic pathways or osteoclast signaling pathways such as PI3K/Akt, IFNγ, TGFβ, and c-Fos (Nguyen et al., 2021).

## 5 Conclusion

PDB has long been considered the second most common disorder of bone metabolism after osteoporosis. However, a dramatic and progressive decline in its prevalence and severity has been observed in many countries, which have made PDB a less frequently diagnosed disease (Michou and Orcel, 2019). The reason for this phenomenon remains unknown, although the rapidity of change observed in geographical areas at elevated PDB prevalence, such as UK, points to an alteration in one or more environmental determinants (Cooper et al., 1999). Moreover, PDB is also considered a model of high turnover bone disease, mainly related to an increase in size and number of acting resorbing osteoclasts at the affected skeletal sites.

Since it first description 1876 either genetic or environmental factors have been implicated in the pathogenesis of this disorder. In the past two decades, thanks to the development in technology, relevant advances have been made in the pathogenesis of PDB, leading to the identification of genetic mutations or variants predisposing to the disease and allowing a better characterization of the role of environmental triggers, such as MV infection, through the development of specifically designed mice models. An update on the pathogenetic mechanisms of PDB and their potential interactions is given on Figure 1. However, despite this remarkable progresses, the molecular and cellular mechanisms leading to the development of this disorder remain in large part unknown. Hopefully, further developments in technology and more detailed studies in animal models and large cohorts of patients will cast additional light into disease mechanism over the next years. There are however important clinical implications relating to the experimental investigations carried out to date. In fact, it has been shown that patients who possess *SQSTM1* gene mutations or other mutations (e.g., *ZNF687* or *PFN1*) have a more severe form of the disease and might thus require more aggressive and continuous treatment. Moreover, the extension of genetic analysis to the relatives of these patients could also allow the identification of new cases of recent onset (even before deformities and other complications have developed) or even allow preventive treatment before the onset of pagetic lesions. Such hypothesis will be verified in the next few years by an international clinical trial in asymptomatic *SQSTM1* mutation carriers (Cronin et al., 2019; Porteous et al., 2020).

Intriguingly, a significant number of genes involved in the pathogenesis of PDB (namely *SQSTM1*, *VCP*, *PFN1*, and *OPTN*) have been also associated with neurodegenerative disorders (e.g., ALS or frontotemporal dementia) suggesting shared pathophysiological mechanisms. Indeed, the *OPTN* gene has been reported to originate from gene duplication of the NFκB regulator named NFκB essential modulator (NEMO), containing also two ubiquitin-binding motifs, all features shared with *SQSTM1* gene. (Tumbarello et al., 2015). Thus, *OPTN* mutations may contribute to both abnormal autophagy and vesicles trafficking, as reported in the ALS-related forms (Ryan and Tumbarello, 2018), and abnormal NFκB activation, as a possible cause of uncontrolled maturation and activation of pagetic osteoclasts. Similarly to *OPTN* and *SQSTM1*, also *VCP* and *PFN1* share common roles in autophagic degradation raising the hypothesis of a common pathogenic mechanism affecting the formation and clearance of misfolded proteins in PDB, ALS and, more generally, in lysosomal storage diseases (Hocking et al., 2012; Lieberman et al., 2012; Vallet and Ralston, 2016b). Indeed, all these genes predispose to a group of neurological conditions included among the MPS category, characterized by accumulation of abnormal protein aggregates within cells, either because the disease-causing mutations result in defects of protein degradation through the proteasome or because they act directly to increase the propensity of the mutated protein to form aggregates (Taylor, 2015). It remains unclear why some patients with these mutations develop PDB while others develop neurological or muscle disorders, but it is likely that the co-inheritance of other variants might affect the clinical phenotype (Ralston, 2020).

Enduring and intriguing mysteries of PDB are also represented by the asymmetric appearance of bone lesions, which are randomly distributed in the body, as well as the non-appearance of new bone lesions during the clinical course of the disease. In this respect, the findings from Diboun et al. (2021), demonstrating significant differentially DNA methylated sites within or near genes regulating osteoclast differentiation, or the response to viral infection and to mechanical loading, might in part explain the clinically anarchic phenotype of PDB.
